# Determination of Histamine in Tuna Fish by a Rapid and Green DART‐TQ MS Method

**DOI:** 10.1002/jms.70027

**Published:** 2026-01-12

**Authors:** Anna Luparelli, William Matteo Schirinzi, Laura Quintieri, Alexandre Verdu, Linda Monaci

**Affiliations:** ^1^ Institute of Biomembranes, Bioenergetics and Molecular Biotechnologies National Research Council (IBIOM‐CNR) Bari Italy; ^2^ Institute of Sciences of Food Production National Research Council (ISPA‐CNR) Bari Italy; ^3^ Applied Mass Spectrometry Bruker France S.A.S. Wissembourg Cedex France

## Abstract

This application note describes the development of a rapid and sustainable screening method for the detection of histamine (HIS) in food, representing a key biogenic amine and safety marker in tuna, based on the coupling between an ambient soft ionization source and a triple quadrupole mass spectrometer (DART–TQ‐MS/MS). The method requires minimal sample preparation and enables the targeted quantification of HIS through optimized MRM transitions (112.2 → 95.0 and 112.2 → 68.1 m/z). The DART–TQ‐MS/MS platform offers an efficient first‐line tool providing rapid preliminary control in complex food matrices before confirmatory analysis.

## 1 Introduction

Biogenic amines (BAs) are low‐molecular‐weight compounds formed by the microbial decarboxylation of amino acids and are widely recognized as indicators of freshness and safety in fish and fishery products [1, 2, 3]. Their formation is influenced by several factors, including harvesting conditions, handling practices, and on‐board processing, which can reflect microbial activity and serve as markers of hygienic quality along the supply chain. Histamine (HIS), a BA, is the primary agent responsible for scombrotoxin fish poisoning, associated with fish consumption and characterized by symptoms that mimic IgE‐mediated allergic reactions. This condition is most linked to scombroid fish, particularly dark‐meat species such as tuna, in which HIS accumulates as a result of bacterial decarboxylation of the amino acid histidine [1]. Although HIS is not harmful at low levels, excessive intake can lead to adverse health effects such as headaches, nausea, and vomiting. In European Union, to ensure consumer protection, Commission Regulation (EC) No. 2073/2005, amended by No. 1019/2013 [2], establishes microbiological criteria for HIS in fishery products. The regulation defines a maximum limit (M) of 200 mg/kg, above which the entire batch is deemed non‐compliant. A lower threshold (m) of 100 mg/kg serves as an alert level; if exceeded in initial samples, additional testing on eight further units is mandated to statistically assess batch compliance [2]. The Food Safety Authority of Ireland (FSAI) reports that most cases of illness are caused by HIS in fish above 200 mg/kg, and often even above 500 mg/kg. In the United States, the Food and Drug Administration (FDA) has set a defect action level for HIS in fish at 50 mg per 100 g. This threshold serves as an indicator of spoilage; concentrations above 500 mg/kg are considered hazardous and associated with scombroid poisoning [4, 5].

HIS quantification in fish and fishery products is crucial for food safety monitoring. Traditional official methods, as described in ISO 19343:2017 [6], primarily rely on chromatographic techniques such as high‐performance liquid chromatography (HPLC) with fluorescence or UV detection, offering high sensitivity and specificity [7]. The 2014 JRC report evaluated the equivalence of HIS determination methods in fishery products by comparing the EU's official HPLC‐UV method with the AOAC Official Method 977.13 [8], a fluorometric method recommended by Codex Alimentarius [9]. While both methods are valid and suitable for regulatory use, they are not fully equivalent, differing in sensitivity and reproducibility. The EU HPLC‐UV method remains the regulatory standard in Europe, whereas the Codex method is widely accepted internationally [9]. The report emphasizes the need for thorough validation and quality control to ensure reliable HIS detection.

While both official methods are robust and validated, their reliance on derivatization and lengthy analysis times drives the development of faster, simpler techniques without sacrificing sensitivity or specificity.

Recent developments in HIS analysis include advanced immunoassays and biosensors that provide rapid, sensitive, and reliable detection. Indirect competitive ELISA methods, such as those using novel enzyme mimics like iron‐cobalt co‐doped carbon dots, offer low detection limits (~0.5 mg/kg) and accurate quantification in fish samples [10]. Commercial ELISA kits (e.g., HistaSure) are validated for regulatory use, showing strong correlation with official chromatographic methods and delivering fast results with good sensitivity and specificity [11]. Additionally, enzymatic biosensors like Biofish‐300 HIS enable very rapid quantification (2–3 min) with high selectivity, supporting real‐time food safety monitoring [12]. These emerging approaches complement traditional methods by reducing analysis time and simplifying sample preparation, although broader regulatory acceptance is still needed.

Beyond conventional ELISA and enzymatic biosensors, several advanced techniques based on ambient mass spectrometry (AMS) are gaining attraction for the assessment of quality and safety in fishery products [13, 14, 15, 16]. AMS encompasses techniques such as desorption electrospray ionization (DESI) and direct analysis in real time mass spectrometry (DART‐MS), which share a key feature: Samples are analyzed enabling direct ionization under ambient conditions, without requiring complex preparation steps or chromatographic separation. These emerging techniques allow in situ, high‐throughput, and real‐time analysis of solids, liquids, and gases, with minimal or no additional sample preparation, and they preserve sample integrity while minimizing in‐source fragmentation [17]. In DART‐MS, the sample is exposed to a stream of heated ionizing gas—typically helium or nitrogen—that facilitates desorption and direct ionization of analytes. This makes DART‐MS particularly suited for rapid screening applications across diverse fields including forensic science [18], food safety and authenticity [18, 19, 20, 21, 22], pharmacology [23, 24], and environmental monitoring [25, 26].

A key innovation of DART‐MS lies in its ability to perform both qualitative and quantitative analyses without the need for chromatographic separation, thereby significantly reducing analysis time, solvent waste, and improving overall operational efficiency.

In the literature, DART is more frequently coupled with high‐resolution mass spectrometry (HRMS) than with low‐resolution mass spectrometry, largely because HRMS enables full‐scan acquisition, accurate mass measurements, and untargeted screening. These features make HRMS particularly well suited for exploratory studies and the analysis of complex food matrices [13, 14, 15, 16]. Nonetheless, DART coupled with triple quadrupole mass spectrometry (DART‐TQ MS) is increasingly gaining attention for targeted quantitative applications, where higher sensitivity, selectivity, and robust quantification are required for routine safety and quality assessment. When integrated with a triple quadrupole mass spectrometer, DART‐MS operates in multiple reaction monitoring (MRM) mode, providing high selectivity and sensitivity even in complex food matrices. This setup allows for the acquisition of informative mass spectra in less than 30 s per sample, positioning DART‐TQ MS as a powerful and green platform for rapid and reliable food safety preliminary assessments before confirmatory analysis. A method previously developed by our team using DART‐TQ‐EVOQ, aimed at protecting saffron authenticity [20], was already evaluated for its environmental sustainability using the Analytical GREEnness calculator (AGREE) (Version v0.5 beta). That assessment reported an AGREE score of 0.88, indicating excellent adherence to the principles of Green Analytical Chemistry and demonstrating that DART‐MS/MS–based workflows can offer both high sustainability and rapid analytical performance with minimal environmental impact [20].

This application note presents the development and implementation of a fast, robust, and sustainable method for the targeted quantification of elevated or potentially hazardous HIS levels in commercial tuna fillets.

## 2 Experimental

### 2.1 Chemicals and Materials

The following solvents and reagents were purchased from Sigma‐Aldrich (Milan, Italy): methanol for HPLC (≥ 99.9%, CAS: 67‐56‐1), hydrochloric acid (HCl, 37%, ACS reagent grade, CAS 7647‐01‐0) and HIS (≥ 97% purity, catalog number H7125, CAS 51‐45‐6) used as analytical standard for calibration curve preparation and spiking experiments. PTFE syringe filters (0.45 μm pore size) were purchased from Sartorius Italy S.r.l. (Varedo, Monza e Brianza, Italy). QuickStrip HTS cards (96‐well format) for DART‐MS analysis were purchased from Bruker Daltonics (Bremen, Germany). Commercial tuna fillets in glass jars were purchased from local supermarkets in Italy. They were stored at room temperature prior to opening and then kept at 4°C for interday repeatability experiments.

### 2.2 Sample Preparation

Extraction was performed by adding 5 mL of cold 100% methanol containing 0.1 M HCl (pre‐chilled at −20°C) to 1 g of commercial tuna fillets. Samples were homogenized on ice using a T 18 digital IKA ULTRA‐TURRAX Ident No. 0003720000 at 15 000 rpm for 60 s. After homogenization, the samples were incubated at −20°C for 30 min and centrifuged at 6500 rpm for 15 min at 4°C. The supernatants were then collected and filtered through 0.45 μm PTFE syringe filters. An aliquot of 1 mL of the filtered extract was evaporated to dryness under a stream of nitrogen and reconstituted in 200 μL of MeOH/H_2_O (80:20, v/v) containing 0.1 M HCl to achieve pre‐concentration and eliminate the initial dilution factor. The reconstituted extracts were finally centrifuged at 13 000 rpm for 5 min to remove any remaining particulates before DART‐MS analysis. A final volume of 2 μL of each extract was deposited onto the DART 96‐well sampling card and allowed to dry at room temperature for 10 min prior to DART‐MS analysis. Preliminary investigations using HIS standard powder enabled the identification of characteristic marker ions, which were subsequently confirmed within the tuna fillet matrix.

### 2.3 Preparation of Calibration Curves Using HIS Standard and Spiked Tuna Fillets

Stock solution of HIS (1000 mg/L) was prepared by dissolving 1 mg of the analytical standard in 1 mL of a MeOH/H_2_O mixture (80:20, v/v). To generate the calibration curve, the HIS stock solution (1000 mg/L) was diluted with MeOH/H_2_O (80:20, v/v) to obtain standard solutions at final concentrations of 200, 400, 800, and 1000 mg/L.

Following preliminary analyses, a matrix‐matched calibration curve was established using commercial tuna fillets. Extracts from blank tuna samples were spiked with HIS at concentrations of 200, 400, 800, and 1000 mg/kg to account for potential matrix effects and to enable accurate quantification in the complex food matrix.

### 2.4 DART‐MS Analysis

All analyses were performed using an EVOQ DART‐TQ^+^ mass spectrometer (Bruker Daltonics GmbH & Co. KG, Fahrenheitstraße 4, 28 359 Bremen, Germany), which combines direct analysis in real time (DART) ionization with triple quadrupole mass spectrometry for high‐throughput, selective, and sensitive quantification. The following workflow was initially applied to HIS standard and subsequently implemented on the complex matrix of commercial tuna fillets. The approach was inspired by a previously published workflow developed for the detection of adulterants in saffron [16].

#### 2.4.1 DART Sample Introduction

Circular scanning mode was selected for its superior reproducibility among the three tested (linear, circular, jumpshot). Optimal ionization efficiency and sensitivity were achieved by experimentally adjusting key parameters: motor speed (0.25 mm/s), scan diameter (2 mm), cone voltage (40 V), cone temperature (350°C), DART temperature (300°C), and gas pressure (20 PSI). Temperature settings were optimized to balance ionization performance with minimal thermal degradation, while the gas flow program (6 s) was fine‐tuned to enhance signal intensity without excessive fragmentation.

#### 2.4.2 Full MS

Full MS scan experiments were carried out in positive ion mode. The MS settings were as follows: scan mode: Q1MS; polarity: positive; Q1 first mass: 100; Q1 last mass: 600; Q1 resolution: 0.7.

#### 2.4.3 Precursor Ion Monitoring

Following full MS analysis, the precursor ion at m/z 112.2 and monitored as follows: scan mode: precursor; polarity: positive; Q1 and Q3 resolution: 1.

#### 2.4.4 Product Ion Scan

Collision energy (CE) was optimized by testing different values to maximize the intensity of fragment ions from the selected precursor. The MS settings were as follows: scan mode: product; polarity: positive; Q1 and Q3 resolution: 2; CE (eV): tested 15, 20, and 25.

#### 2.4.5 MRM

Experiments were carried out to optimize the final conditions for selecting stable and sensitive transitions and corresponding fragment ions, which would be used as quantitative and qualitative markers for HIS detection in tuna samples. The MS settings were as follows: scan mode: MRM; polarity: positive; Q1 [112.2] and Q3 [95.0], [68.1]; Q1 and Q3 resolution: 2; CE (eV): 20.

## 3 Results and Discussion

### 3.1 Preliminary Assessment of HIS Standard and Construction of Calibration Curve

Full‐scan MS experiments were conducted in the m/z range 100–600 on a HIS standard to establish a comprehensive ion profile. This initial screening enabled the identification of the most intense and stable precursor ion at m/z 112.2, which was selected for subsequent fragmentation studies. Product ion monitoring experiments were then performed to optimize ce values. Among the tested settings (15, 20, and 25 eV), 20 eV provided the best balance between fragment ion intensity and signal stability, as evidenced by consistent precursor‐to‐product ion ratios across replicate injections. This optimization step led to the identification of two major product ions at m/z 95.0 (quantifier) and 68.1 (qualifier), selected for their high abundance and reproducibility. These transitions were ultimately used in MRM mode, ensuring sensitive and selective quantification of HIS in complex food matrices, such as commercial tuna fillets.

**FIGURE 1:**
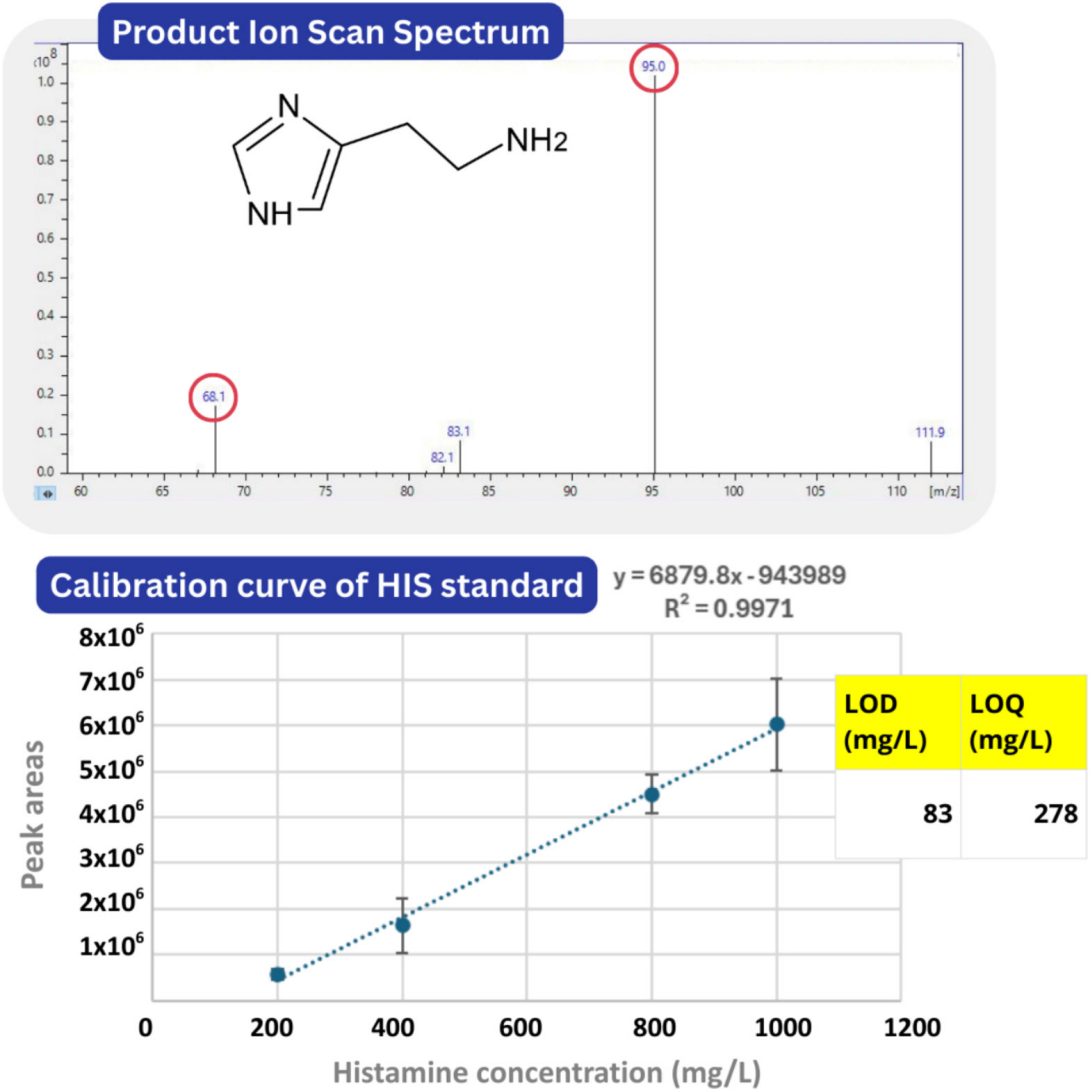
Top: product ion spectrum of HIS standard (precursor m/z 112.2) showing the main fragments at m/z 95.0 (quantifier) and 68.1 (qualifier). Bottom: calibration curve constructed using DART‐TQ MS from HIS standard solutions (200–1000 mg/L), with a regression equation of *y* = 6879.8*x* − 943 989 and excellent linearity (*R* ^2^ = 0.9971). Calculated LOD and LOQ were 83 and 278 mg/L, respectively.

A calibration curve for HIS quantification was constructed from a 1000 mg/L stock solution in MeOH/H_2_O (80:20, v/v), previously described. Serial dilutions were prepared to obtain standard solutions at 200, 400, 800, and 1000 mg/L, each analyzed in quadruplicate. The curve was built by plotting the peak area of the transition m/z 112.2 → 95 against the corresponding concentrations. The resulting regression equation was *y* = 6879.8*x* − 943 989 with *R* ^2^ = 0.9971, indicating excellent linearity. The method's LOD and LOQ, estimated at signal‐to‐noise ratios of 3:1 and 10:1, respectively, were 83.3 and 277.7 mg/L. The coefficient of variation (CV%) of peak areas remained consistently below 22%, confirming good repeatability. These results demonstrate that the method is reliable for quantitative analysis of HIS in standard solutions, with high sensitivity and reproducibility. Figure 1 summarizes the key steps and results described above.

### 3.2 Production of Matrix‐Matched Calibration Curves in Tuna Fillets and Recovery Assessment

**FIGURE 2:**
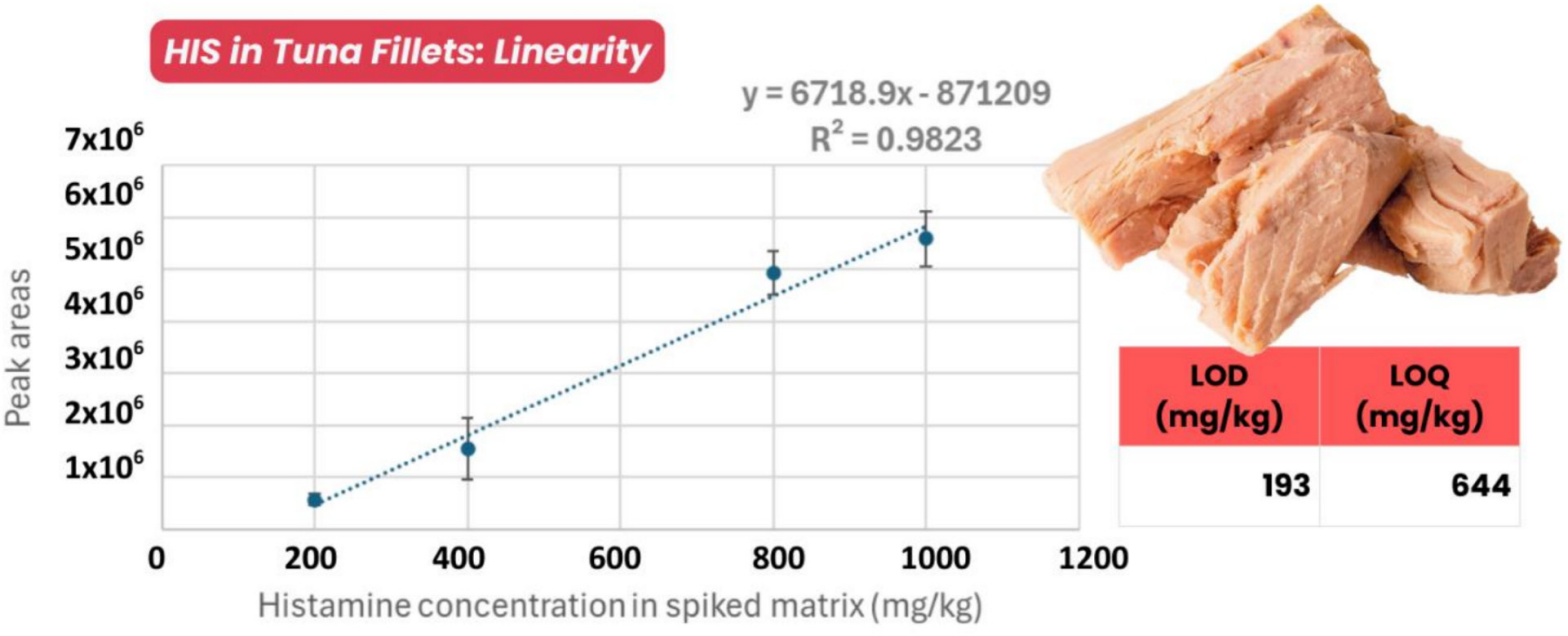
Matrix‐matched calibration curve for HIS quantification in tuna extracts. The calibration curve was constructed by spiking blank tuna matrix extracts with HIS at four concentration levels (200, 400, 800, and 1000 mg/kg).

To evaluate the analytical performance of the method in real samples, a matrix‐matched calibration curve was prepared using commercial tuna fillets. The tuna samples were extracted and processed as previously described. A HIS stock solution (1000 mg/L), prepared in MeOH/H_2_O (80:20, v/v), was serially diluted to obtain standard solutions at concentrations of 200, 400, 800, and 1000 mg/L. Blank tuna matrix extracts were spiked with HIS at four selected concentration levels and analyzed in quadruplicate by DART‐MS/MS using an MRM method optimized on the pure standard and validated on the real matrix. This validation ensured the absence of interfering signals with the target precursor ion. The resulting calibration curve demonstrated good linearity over the tested range, with a coefficient of determination (*R* ^2^) of 0.9823, confirming a consistent correlation between HIS concentration and peak area (Figure 2). The calculated limit of detection (LOD) and limit of quantification (LOQ) were 193 and 644 mg/kg, respectively. These values are higher than those typically obtained in solvent, indicating a pronounced matrix effect that reduced the sensitivity of the method. This highlights the necessity of performing matrix‐matched calibration to ensure accurate quantification of HIS in complex food matrices such as tuna. In order to improve the sensitivity of the developed method, future efforts might be directed at introducing a pre‐purification/preconcentration step by SPE clean up step in order to reduce the matrix effect that might impact the overall sensitivity of the selected markers.

To assess method recovery, a dedicated experiment was performed by spiking tuna samples with HIS standard before the extraction process at a fixed concentration of 1000 mg/kg. Three extraction replicates of these pre‐extraction spiked samples were prepared and compared with blank tuna extracts spiked with HIS at 1000 mg/kg (post‐extraction spike). Each sample was analyzed in five replicates to ensure repeatability and reliability of the results. The recovery was calculated by comparing the measured HIS concentration in the spiked samples before extraction with that of the post‐extraction spiked samples, expressed as a percentage. The recoveries obtained in the three replicates were 55 ± 8%. Such recovery value could be due to several reasons such as the matrix effect that might interfere with analyte ionization or detection, overloading of the extraction solution or adsorption of the analytes to the vials.

As far as method precision is concerned, it was assessed by calculating the intraday CV% on a tuna sample spiked at 1000 mg/kg of HIS, analyzed within a single day (Day 1). Interday precision was evaluated by analyzing the same spiked sample over three consecutive days (Days 1, 2, and 3), and the corresponding CV% was calculated across all measurements. The intraday CV% was determined to be 23%, while the interday CV% was 35%. Throughout the analysis period, the sample was stored at 4°C to preserve its stability.

Despite some challenges, the ongoing efforts directed to exploit the strengths of DART‐MS/MS shade light on its promising future for food analysis. Indeed, DART‐MS offers extremely rapid analysis, minimal sample preparation, and high throughput, making it particularly suitable for preliminary screening by food industries. Ambient ionization enables direct analysis of solid or semi‐solid samples, reducing the time for extraction steps and the risk of analyte loss. However, quantitative performance can be limited by matrix effects, lower ionization efficiency compared to LC–MS/MS, and reduced sensitivity for specific compounds. The lack of chromatographic separation may lead to signal suppression or interferences that might affect method sensitivity; also, reproducibility can be affected by sample positioning and DART source parameters. The inclusion of an isotopically labelled internal standard will allow to correct for any ion suppression and matrix effect and will be taken into consideration for future studies. In addition, advances in MS instrumentation, new food‐customized databases and advanced data analysis tools are expected to improve throughput, automation, and analytical reliability, enabling further applied studies on the analysis of toxic compounds in foods across different types of matrices.

## 4 Conclusions

In conclusion, the developed DART‐TQ‐MS method demonstrated satisfactory performance for the quantification of HIS in tuna, with a matrix‐matched calibration curve showing good linearity (*R* ^2^  =  0.9823) in the range of 200 to 1000 mg/kg which covers the concentration range of interest for potentially hazardous HIS levels, according to what was reported by the FSAI that reports most cases of illness caused by HIS in fish above 200 mg/kg. The recovery was 55 ± 8% and intraday and interday precision were respectively 23% and 35%, with LODs and LOQs of 193 and 644 mg/kg, highlighting areas for further optimization. As reported by the FDA, concentrations above 500 mg/kg are considered hazardous for consumers since they are associated with severe scombroid poisoning, while the method devised in this application note could be successfully applied to detect hazardous concentrations of this BA in this matrix. On the other hand, current regulatory limits for HIS in fish products typically range from 100 to 200 mg/kg as indicators (e.g., FDA limit of 50 mg/100 g and EU limit of 100 mg/kg), underscoring the importance of sensitive and accurate detection methods. The method shows to be promising for rapid and reliable screening of levels of HIS in tuna fish, considered a critical BA regulated by food safety authorities. Further validation across diverse food matrices is required to confirm a broader applicability. Although the developed method demonstrates robust and satisfactory performance for rapid screening, achieving regulatory‐level sensitivity would require further optimization. Overall, DART‐TQ‐MS represents a valuable tool for fast screening in food safety and authenticity control, with potential extension to other BA contaminants and residues.

## Data Availability

The data that support the findings of this study are available from the corresponding author upon reasonable request.
